# Cytogenetics of the small-sized fish, *Copeina guttata* (Characiformes, Lebiasinidae): Novel insights into the karyotype differentiation of the family

**DOI:** 10.1371/journal.pone.0226746

**Published:** 2019-12-19

**Authors:** Gustavo Akira Toma, Renata Luiza Rosa de Moraes, Francisco de Menezes Cavalcante Sassi, Luiz Antonio Carlos Bertollo, Ezequiel Aguiar de Oliveira, Petr Rab, Alexandr Sember, Thomas Liehr, Terumi Hatanaka, Patrik Ferreira Viana, Manoela Maria Ferreira Marinho, Eliana Feldberg, Marcelo de Bello Cioffi

**Affiliations:** 1 Laboratório de Citogenética de Peixes, Departamento de Genética e Evolução, Universidade Federal de São Carlos, São Carlos, São Paulo, Brazil; 2 Secretaria de Estado de Educação de Mato Grosso, Cuiabá, Mato Grosso, Brazil; 3 Laboratory of Fish Genetics, Institute of Animal Physiology and Genetics, Czech Academy of Sciences, Liběchov, Czech Republic; 4 Institute of Human Genetics, University Hospital Jena, Jena, Germany; 5 Instituto Nacional de Pesquisas da Amazônia, Manaus, Amazonas, Brazil; 6 Museu de Zoologia da Universidade de São Paulo, São Paulo, São Paulo, Brazil; China University of Science and Technology, CHINA

## Abstract

Lebiasinidae is a small fish family composed by miniature to small-sized fishes with few cytogenetic data (most of them limited to descriptions of diploid chromosome numbers), thus preventing any evolutionary comparative studies at the chromosomal level. In the present study, we are providing, the first cytogenetic data for the red spotted tetra, *Copeina guttata*, including the standard karyotype, C-banding, repetitive DNA mapping by fluorescence *in situ* hybridization (FISH) and comparative genomic hybridization (CGH), providing chromosomal patterns and novel insights into the karyotype differentiation of the family. Males and females share diploid chromosome number 2n = 42 and karyotype composed of 2 metacentric (m), 4 submetacentric (sm) and 36 subtelocentric to acrocentric (st-a) chromosomes. Blocks of constitutive heterochromatin were observed in the centromeric and interstitial regions of several chromosomes, in addition to a remarkably large distal block, heteromorphic in size, which fully corresponded with the 18S rDNA sites in the fourth chromosomal pair. This overlap was confirmed by 5S/18S rDNA dual-color FISH. On the other hand, 5S rDNA clusters were situated in the long and short arms of the 2^nd^ and 15^th^ pairs, respectively. No sex-linked karyotype differences were revealed by male/female CGH experiments. The genomic probes from other two lebiasinid species, *Lebiasina melanoguttata* and *Pyrrhulina brevis*, showed positive hybridization signals only in the NOR region in the genome of *C*. *guttata*. We demonstrated that karyotype diversification in lebiasinids was accompanied by a series of structural and numeric chromosome rearrangements of different types, including particularly fusions and fissions.

## Introduction

The Neotropical freshwater ichthyofauna comprises approximately 16% of the worldwide fish biodiversity, encompassing about 5,200 presently recognized species in 17 orders [1,2]. However, this number is underestimated, as a steadily growing number of studies points on previously overlooked cases of cryptic species and species complexes (e.g., [3–7]). In this context, the contribution of the cytogenetic studies to the knowledge of biodiversity and evolution of the Neotropical fishes is remarkable, providing useful taxonomic and evolutionary data (reviewed in [8]). Additionally, methodological advances in molecular cytogenetics, namely diverse variants of fluorescence *in situ* hybridization (FISH), allow to decipher karyotype/genome evolution among related species, including the degree of preserved conserved synteny and the characterization of structural and functional organization of genomes [9–20]. These approaches already helped to document cryptic species diversification [21–29] as well as to track remarkable karyotype stability [30,31], the response of the genome dynamics to environmental cues [32] or its correlation with geographic distribution [33].

Within this enormous diversity of Neotropical ichthyofauna, Lebiasinidae contains miniature to small-sized fishes (1.6–7.0 cm) distributed throughout small streams of Central (Panamá and Costa Rica) and South America, except for Chile [34,35]. The family comprises seven genera and about 74 species, distributed in two subfamilies: Lebiasininae, with the *Derhamia*, *Lebiasina* and *Piabucina*; and more diverse Pyrrhulininae, with *Copeina*, *Copella*, *Nannostomus* and *Pyrrhulina* [35,36].

For a long time, most cytogenetic data for this group were limited to descriptions of diploid chromosome numbers (2n) [37,38] and/or conventional banding procedures [39,40] **(Table 1).** However, these first data were sufficient enough to evidence that a substantial karyotype diversity does occur in some lebiasinid lineages, namely in the genus *Nannostomus*, where 2n varies from 2n = 22 (in *N*. *unifasciatus*) to 2n = 46 (in *N*. *trifasciatus*), indicating the frequent action of Robertsonian rearrangements [41].

**Table 1:** Updated table of chromosomal data for Lebiasinidae fishes, adapted from [42].

Species	2n (sex)	Karyotype	Reference
**Pyrrhulininae subfamily** ***Copeina***			
*C*. *guttata*	42 (?)	-	[37]
*C*. *guttata*	42♂♀	2m+4sm+36st/a	**Present study**
***Copella***			
*C*. *arnoldi*	44 (?)	-	[37]
*C*. *nattereri*	36 (?)	-	[37]
*Copella* sp.	26 (?)	-	[37]
*Copella* sp.	24 (?)	-	[37]
***Nannostomus***			
*N*. *beckfordi* (A)	42 ♂	2m+40a	[38]
*N*. *beckfordi* (B)	44 (?)	-	[37]
*N*. *beckfordi* (C)	36 (?)	-	[37]
*N*. *eques* (A)	34 (?)	34a	[38]
*N*. *eques* (B)	36 (?)	-	[37]
*N*. *arrisoni*	40 (?)	-	[37]
*N*. *marginatus*	42 (?)	-	[37]
*N*. *trifasciatus* (A)	46 (?)	-	[37]
*N*. *trifasciatus* (B)	38 (?)	-	[37]
*N*. *trifasciatus* (C)	30 (?)	-	[37]
*N*. *trifasciatus* (D)	24 (?)	-	[37]
*N*. *unifasciatus*	22 (?)	-	[37]
***Pyrrhulina***			
*Pyrrhulina* cf. *australis*	40♂♀	6st+34a	[39]
*Pyrrhulina* sp.	42 (?)	2m+2sm+38st/a	[40]
*P*. *australis*	40♂♀	4st+36a	[43]
*Pyrrhulina* aff. *australis*	40♂♀	4st+36a	[43]
*P*. *brevis*	42♂♀	2sm + 4st + 36a	[44]
*P*. *semifasciata*	41♂	1m + 4st + 36a ♂	[44]
	42♀	4st + 38a ♀	[44]
**Lebiasininae subfamily** ***Lebiasina***			
*Lebiasina bimaculata*	36♂♀	36m/sm	[42]
*Lebiasina melanoguttata*	36♂♀	36m/sm	[42]

More recently, molecular cytogenetics begun to be implemented in a finer-scale characterization of karyotype structures in certain lebiasinid taxa. More specifically, FISH-based repetitive DNA mapping, comparative genomic hybridization (CGH) and whole chromosome painting (WCP) have been applied in four *Pyrrhulina* [43,44] and in two *Lebiasina* species [42]. *Lebiasina bimaculata* and *L*. *melanoguttata*, the two analyzed species, possess 2n = 36 and the cytogenetic comparison between them and members of the family Ctenoluciidae supported the previous hypothesis of a close relationship between them [42]. On the other hand, with 2n = 40 in *P*. *australis* and *Pyrrhulina* aff. *australis*, 2n = 42 in *P*. *brevis* and 2n = 41/42 in *P*. *semifasciata*, this genus comprises cytogenetic diversity in Lebiasinidae [43,44]. It has been demonstrated that *P*. *australis* and *Pyrrhulina* aff. *australis*, both with 2n = 40, have different karyotype structures, indicating distinct evolutionary units [43]. In addition, *P*. *semifasciata* presents a differentiation between males (2n = 41) and females (2n = 42) due to the presence of a multiple sex chromosome system of X_1_X_1_X_2_X_2_/X_1_X_2_Y type, where the large metacentric Y sex chromosome arose from chromosomal fusion in males [44].

The aim of the present study was to extend the knowledge on the trends and underlying mechanisms of karyotype differentiation in Lebiasinidae, by analyzing the karyotype organization of a representative of *Copeina*, a genus not analyzed to date, using both conventional (Giemsa staining and C-banding) and molecular cytogenetic (physical mapping of 5S and 18S rDNA and CGH) procedures. In this sense, this study represents the first one conducted in the genus *Copeina* and is included in a series focusing on the cytogenetics and cytogenomics of Lebiasinidae fishes.

## Material and methods

### Samples

**Fig 1:**
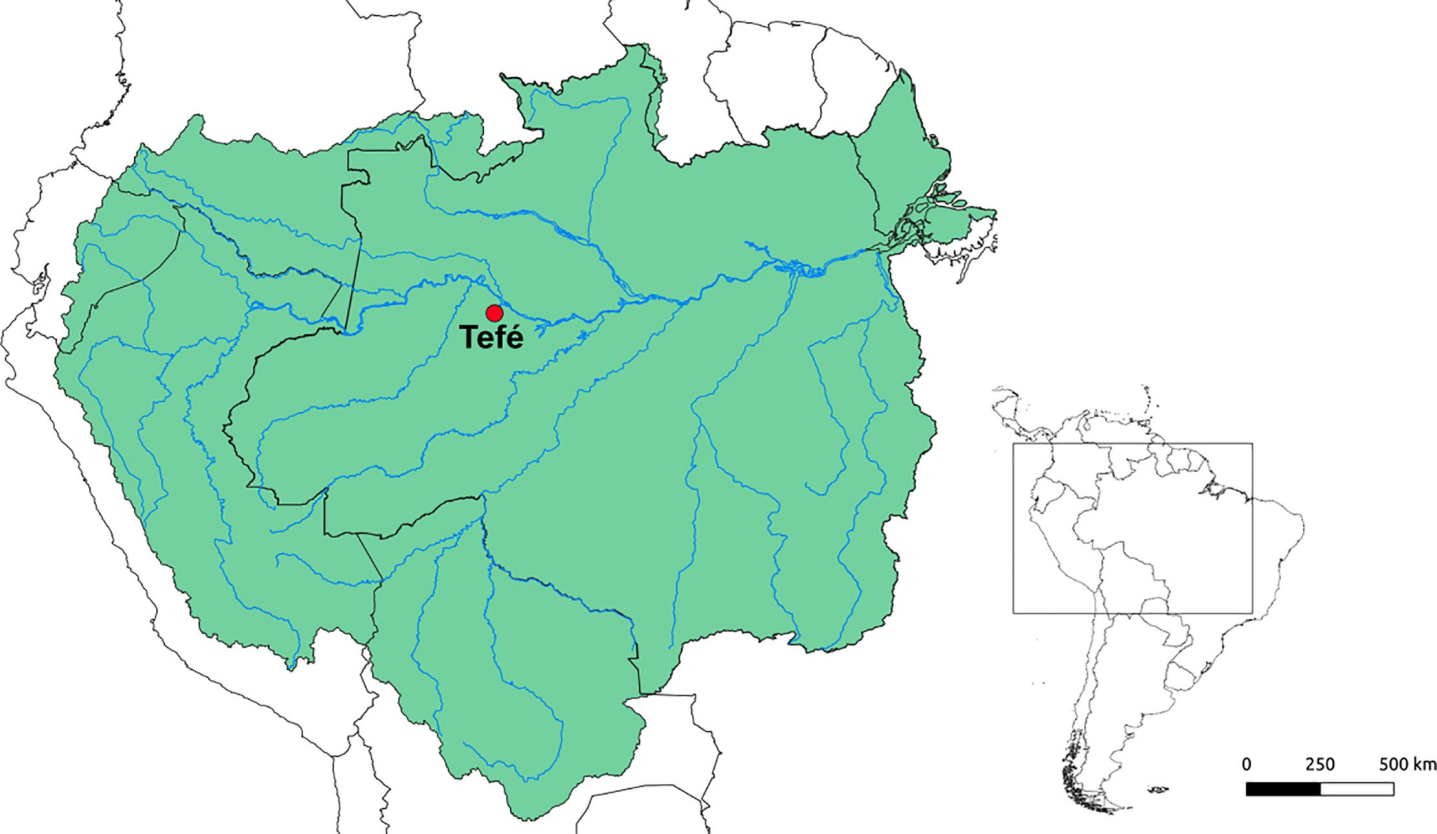
Amazon River basin area (in green), with the red dot indicating the collection site (Tefé, Amazonas state).

Sixteen individuals (11 females and five males) of *Copeina guttata* from the Tefé river (Tefé, AM, Brazil: 3°39'49.5"S; 64°59'40.0"W) were analyzed (**Fig 1**). The fishes were collected with the authorization of the Chico Mendes Institute for Biodiversity Conservation (ICMBIO), System of Authorization and Information about Biodiversity (SISBIO-License No. 48628–2) and National System of Genetic Resource Management and Associated Traditional Knowledge (SISGEN-A96FF09). All individuals were properly identified by morphological criteria, and voucher specimens were deposited in the fish collections of the Museu de Zoologia, Universidade de São Paulo (MZUSP), under the number 124915.

### Chromosome preparation and C-banding

Mitotic chromosomes were obtained from kidney by the air-drying technique [45]. All experiments followed ethical and anesthesia conducts approved by the Ethics Committee on Animal Experimentation of the Universidade Federal de São Carlos (Process number CEUA 1853260315), sacrificing the animals with clove oil (Eugenol) overdosis. Constitutive heterochromatin was detected by C-banding following [46].

### Repetitive DNA mapping with fluorescence *in situ* hybridization (FISH)

Two tandemly arrayed DNA sequences isolated from the genome of *Hoplias malabaricus*, previously cloned into plasmid vectors and propagated in competent cells of *Escherichia coli* DH5α were used as probes (Invitrogen, San Diego, CA, USA). The first probe corresponded to the 5S rRNA coding region, comprising 120 base pairs (bp) associated with a non-transcribed spacer, NTS [47], labeled with the Nick-Translation mix kit (Roche, Manheim, Germany) using the SpectrumOrange-dUTP (Vysis, Downers Grove, IL, USA). The second probe corresponded to a 1,400 bp segment of the 18S rRNA gene [48], also labeled by means of Nick-Translation but using the SpectrumGreen-dUTP (Vysis, Downer Grove, IL, USA). FISH was performed under high stringency conditions following the protocol described in [18].

### Comparative genomic hybridization (CGH)

Two sets of experiments were designed for this study. The first one focused on intraspecific comparisons, i.e., between male and female genomes. For this purpose, genomic DNA (gDNA) from male and female specimens was extracted from liver by the standard phenol-chloroform-isoamylalcohol method [49]. The gDNAs were subsequently differentially labeled either with SpectrumOrange-dUTP or with SpectrumGreen-dUTP (Vysis, Downers Grove, IL, USA) using a Nick-Translation mix kit (Roche, Manheim, Germany). The hybridization procedure was performed according to Yano et al. [18]. The probe mixture per each slide contained 500 ng of male-derived gDNA, 500 ng of female-derived DNA and 15 μg of C0t-1 DNA (corresponding to sex of the investigated specimen) obtained according to Zwick et al. [50]. The probes were precipitated with 100% ethanol and the dry pellets were mixed with a hybridization buffer containing 50% formamide, 2xSSC, 10% SDS, 10% dextran sulfate and Denhardt’s reagens (pH 7.0). Hybridization took place in a moist chamber for 72 h. After post-hybridization washes, chromosomes were counterstained with 4,6-diamidino-2-phenylindole (DAPI) (1.2 μg/ml) and mounted in antifade solution (Vector, Burlingame, CA, USA).

The second set of experiments was focused on interspecific genomic comparisons (Zoo-FISH) between *Copeina*, *Lebiasina* and *Pyrrhulina* species. For this purpose, gDNA either from *L*. *melanoguttata* or *P*. *brevis* was co-hybridized with gDNA of *C*. *guttata* on chromosomes of *C*. *guttata*. In the first assay, 500 ng of female-derived gDNA of *C*. *guttata* labeled with SpectrumGreen-dUTP (Vysis, Downers Grove, IL, USA), 500 ng of female-derived gDNA of *L*. *melanoguttata* labeled with SpectrumOrange-dUTP (Vysis, Downers Grove, IL, USA) and 15 μg of C0t-1 DNA of each species were used to prepare a final probe mixture. In the second assay, 500 ng of female-derived gDNA of *C*. *guttata* labeled with Spectrum Green-dUTP (Vysis, Downers Grove, IL, USA), 500 ng of female-derived gDNA of *P*. *brevis* labeled with Spectrum Orange-dUTP (Vysis, Downers Grove, IL, USA) and 15 μg of C0t-1 DNA of each species were used. The probes were dissolved in the same hybridization buffer and the CGH procedure followed the same protocol as described above.

### Microscopy and image processing

At least 30 metaphase spreads per individual were analyzed to confirm 2n, karyotype structure and FISH results. Images were captured using an Olympus BX50 microscope (Olympus Corporation, Ishikawa, Japan) with CoolSNAP and the images were processed using Image Pro Plus 4.1 software (Media Cybernetics, Silver Spring, MD, USA). Chromosomes were classified as metacentric (m), submetacentric (sm), subtelocentric (st) or acrocentric (a), according to their arm ratios [51].

## Results

### Karyotyping and C-banding

**Fig 2:**
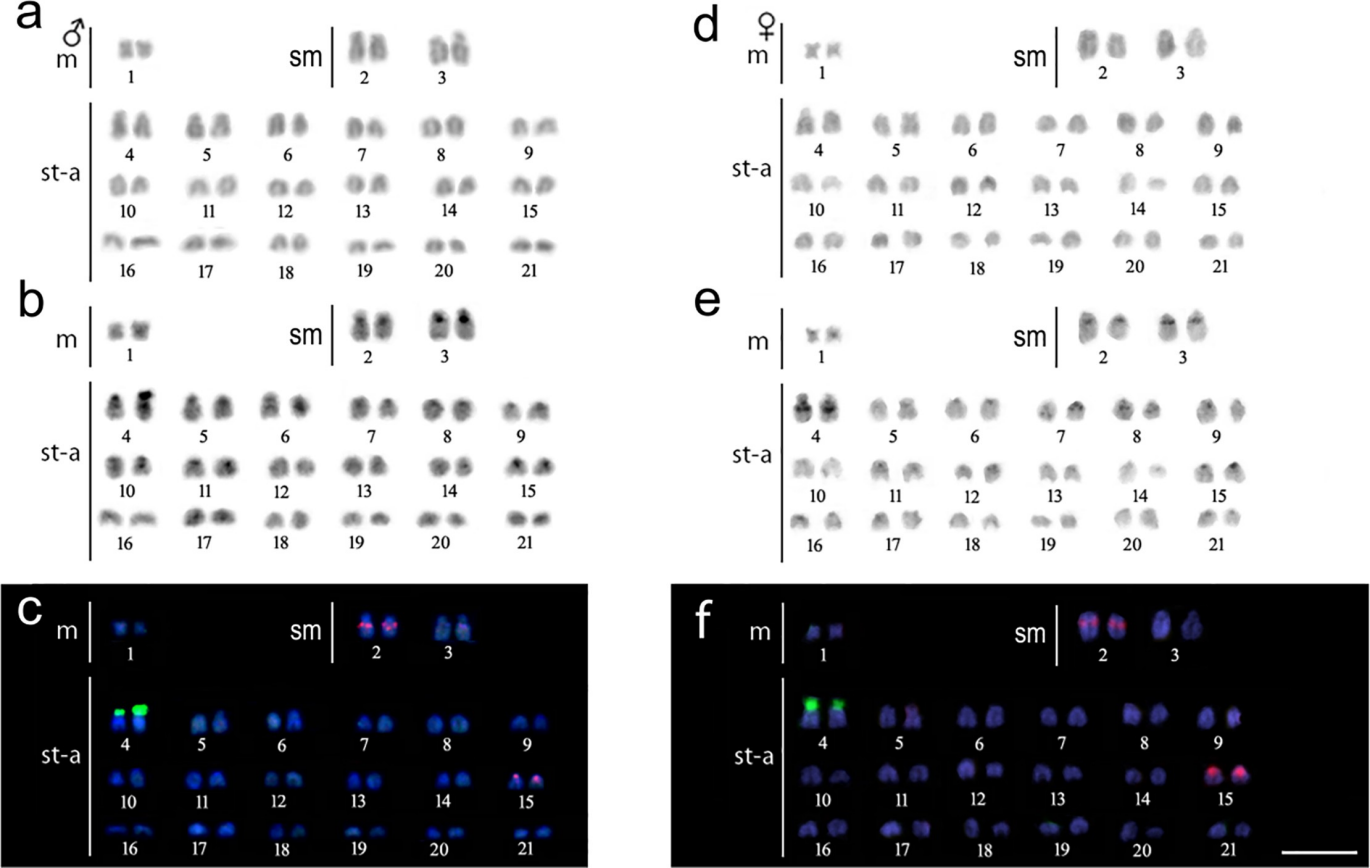
**Karyotypes of *Copeina guttata* male (a, b, c) and female (d, e, f) arranged from Giemsa-stained (a, d), C-banded chromosomes (b, e) and those after dual-color FISH with 18S (green) and 5S (red) rDNA probes (c, f).** Chromosomes were counterstained with DAPI (blue). Scale bar = 5 μm.

The diploid chromosome number of *Copeina guttata* was 2n = 42 and the karyotype was composed of 2m + 4sm + 36st/a chromosomes, both in males (**Fig 2A**) and females (**Fig 2D**). Blocks of constitutive heterochromatin were located in the proximal and interstitial regions of most chromosomes (**Fig 2B and 2E**). A conspicuous heteromorphic block was observed on the 4^th^ chromosomal pair (**Fig 2C**).

### Chromosomal mapping of ribosomal DNAs

Dual-color FISH revealed a single 18S rDNA site covering the entire p arms of a subtelocentric pair Nº. 4. On the other hand, the 5S rDNA loci were located interstitially on the long arms of the pair No. 2 and in the pericentromeric region of the acrocentric pair No. 15 (**Fig 2C and 2F**).

### Comparative genomic hybridization (CGH)

**Fig 3:**
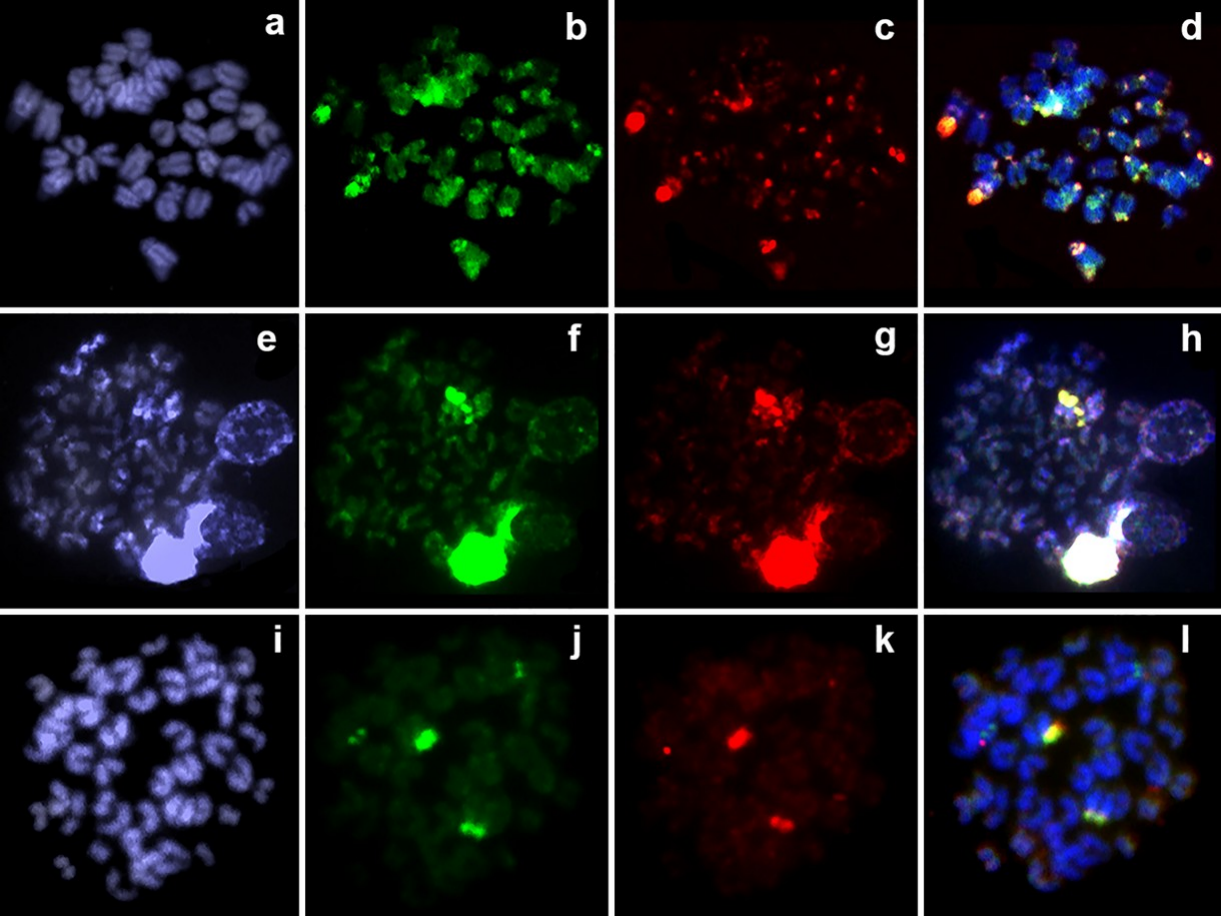
Comparative genomic hybridization (CGH) for intra- and interspecific comparisons in the female metaphase plates of *Copeina guttata*. (**a-d**) Male- and female-derived genomic probes from *C*. *guttata* mapped against female chromosomes of *C*. *guttata* (**e-h**) Female-derived genomic probes from both *Pyrrhulina brevis* and *C*. *guttata* mapped against female chromosomes of *C*. *guttata*. (**i-l**) female-derived genomic probes from *Lebiasina melanoguttata* and *C*. *guttata* hybridized together against female chromosomes of *C*. *guttata*. First column (**a, e, i**) DAPI images (blue); second column (**b, f, j**): hybridization patterns using female gDNA of *C*. *guttata* (**b**), female-derived gDNA of *P*. *brevis* (**f**), and female gDNA of *C*. *guttata* (**j**); third column (**c, g, k**): hybridization patterns using male gDNA of *C*. *guttata* (**C**), female-derived gDNA of *C*. *guttata* (**g**), and female gDNA of *Lebiasina melanoguttata* (**k**). Fourth column (**d, h, l**): merged images of both genomic probes and DAPI staining. The common genomic regions are depicted in yellow.

The intraspecific genomic comparison (**Fig 3A)** between male (**Fig 3B**) and female genomes (**Fig 3C**) revealed in both sexes a hybridization overlap in the centromeric and telomeric regions on almost all chromosomes, a strong binding preference for the 18S rDNA cluster (**Fig 3D**) and no sex-specific sequences accumulations.

To study the degree of genome divergence among selected lebiasinid genera (on the level of repetitive DNA fraction–**Fig 3E and 3I**), gDNA of *Copeina guttata* was compared with other lebiasinids in two sets of experiments. The first assay, which compared gDNA of *Pyrrhulina brevis* (**Fig 3F**) and of *C*. *guttata* (**Fig 3G**) revealed a hybridization pattern in the 18S rDNA region (**Fig 3H**). Similar results were found in the second assay comparing gDNA of *C*. *guttata* (**Fig 3J**) and *Lebiasina melanoguttata* (**Fig 3K**). In both interspecific experiments, also small signals generated by both compared probes were accumulated together in the centromeric regions of some chromosomes.

## Discussion

Lebiasinidae is a relatively large family, which encompasses 74 valid species in seven genera distributed in two subfamilies (Lebiasininae and Pyrrhulininae) [35]. Therefore, the lack of genetic and chromosomal studies for most of its species (**Table 1**) impairs the comparative analyzes to be performed and the main evolutionary trends and chromosomal relationships to be highlighted. Of the seven lebiasinid genera, representatives of only two (*Lebiasina* and *Pyrrhulina*) have been analyzed more thoroughly by us via selected molecular cytogenetic techniques [42–44]. In this paper, we provide the first molecular-cytogenetical approach in *Copeina guttata*, evidencing a karyotype composed of 2n = 42 chromosomes (2m+4sm+36st/a), which fits in the range of 2n already known for Lebiasinidae[41]. Besides that, this karyotype exhibits a predominance of acrocentric chromosomes–another common karyotype feature known for Pyrrhulininae (**Table 1**).

Since 2n = 36 chromosomes can be recognized as the plesiomorphic condition for Lebiasinidae[42], we may hypothesize that karyotype diversification in lebiasinids, particularly in certain taxa of the Pyrrhulininae (for details, see **Table 1**) was accompanied by a series of structural chromosome rearrangements of different types (including particularly fusions and fissions). While *Pyrrhulina*, *Nannostomus* and *Copeina* species analyzed up to now present karyotypes dominated by mono-armed chromosomes, cytogenetic studies conducted in the *Lebiasina* fishes (Lebiasininae) evidenced a contrasting scenario with a divergent karyotype macrostructure, composed only by bi-armed chromosomes **(Table 1).** These two major evolutionary pathways suggest that extensive structural chromosome rearrangements have occurred in the karyotype evolution of this family. In fact, extensive karyotype variability is usually present in fish groups with small isolated populations [52–53], indicating that such variations were fixed and considered as strong evolutionary drivers, facilitating adaptation and/or postzygotic isolation leading even to speciation [54–59].

The 5S and 18S rDNA clusters can be located on a single chromosome pair (i.e. *Lebiasina bimaculata* in [42]), and in two or more chromosomes, as exemplified in *Lebiasina melanoguttata* [42] and *Pyrrhulina* species [43,44]. In this context, *Copeina guttata* also fits into this scenario, with a single chromosomal pair bearing 18S rDNA loci and two pairs bearing 5S rDNA accumulations. Despite the small sampling, a heteromorphic pattern was also observed encountered, particularly in the size of 18S rDNA loci between the homologous chromosomes in males (**Fig 2C**). The potential sex linkage deserves further investigation with a larger sampling. Nonetheless, similar polymorphism is widespread among diverse organisms and it may be caused by i) non-equal crossing over or sister chromatid exchange, ii) subsequent segregation in meiosis and iii) a degree of chromatin condensation inside a given rDNA loci [60–64]. Additionally, the interstitial position of 5S rDNA seems to be conserved in fishes, so as the presence of both ribosomal genes on different chromosomal pairs [65]. On the other hand, 18S rDNA is commonly located in telomeric regions, as also found in *C*. *guttata*, and this location may facilitate a dispersal of this tandem repeat to another chromosomes, in agreement with Rabl’s model (reviewed in [66]).

CGH experiments were performed to track the extent of genome divergence among *Copeina guttata* and other lebiasinids. Besides several pericentromeric and telomeric signals, only a large region of co-hybridization corresponding to the 18S rDNA site (largely conserved in sequence) was encountered, revealing that, at first, *Lebiasina*, *Copeina*, and *Pyrrhulina* species differ profoundly in the composition and distribution of their repetitive sequences. This is not surprising given that certain repetitive DNA classes such as satellite DNAs and transposable elements display rapid rate of evolution that generates often new species-specific repeat variants (e.g. [67]). CGH focusing on male and female comparison revealed no differences in repetitive DNA accumulations in either sex, suggesting either the absence of a sex-chromosome system in this species or its cryptic nature that might escape recognition due to the limitations in resolution of the method. It would be not surprising as morphologically undistinguishable sex chromosomes may appear in fish lineages where otherwise taxa with multiple sex chromosomes occasionally appear (for example, in annual killifishes of the genus *Nothobranchius*; [68,69]). In fact, till now only *P*. *semifasciata* possesses a well differentiated sex chromosome system [44], besides some indication for a probable ZZ/ZW system in *Lebiasina bimaculata* [42] and XX/XY in closely related members of Ctenoluciidae [70].

In conclusion, our study is the first one to offer reliable chromosomal data for *C*. *gutatta* by both conventional and molecular cytogenetic protocols, despite the small size of this and related species make these attempts notoriously difficult. Our data supports the likely proximity of Pyrrhulininae species (*Pyrrhulina*, *Nannostomus* and *Copeina)* in contrast with Lebiasininae ones, due to remarkable variation in their karyotype organization. Besides, data from comparative genomic hybridization experiments also highlighted an advanced stage of sequence divergence, evidencing their evolutionary diversification. This is part of a series of cytogenetic and cytogenomic studies on Lebiasinidae fishes, aiming to comprehensively examine the chromosomal evolution of these miniature fishes.
